# Theoretical analyses on water cluster structures in polymer electrolyte membrane by using dissipative particle dynamics simulations with fragment molecular orbital based effective parameters†

**DOI:** 10.1039/c8ra07428c

**Published:** 2018-10-08

**Authors:** Koji Okuwaki, Yuji Mochizuki, Hideo Doi, Shutaro Kawada, Taku Ozawa, Kenji Yasuoka

**Affiliations:** Department of Chemistry and Research Center for Smart Molecules, Faculty of Science, Rikkyo University 3-34-1 Nishi-ikebukuro Toshima-ku Tokyo 171-8 Japan fullmoon@rikkyo.ac.jp okuwaki@rikkyo.ac.jp; Institute of Industrial Science, The University of Tokyo 4-6-1 Komaba Meguro-ku Tokyo 153-8505 Japan; JSOL Corporation 2-5-24 Harumi Chuo-ku Tokyo 104-0053 Japan; Department of Mechanical Engineering, Keio University Yokohama 223-8522 Japan

## Abstract

The mesoscopic structures of polymer electrolyte membrane (PEM) affect the performances of fuel cells. Nafion® with the Teflon® backbone has been the most widely used of all PEMs, but sulfonated poly-ether ether-ketone (SPEEK) having an aromatic backbone has drawn interest as an alternative to Nafion. In the present study, a series of dissipative particle dynamics (DPD) simulations were performed to compare Nafion and SPEEK. These PEM polymers were modeled by connected particles corresponding to the hydrophobic backbone and the hydrophilic moiety of sulfonic acid group. The water particle interacting with Nafion particles was prepared as well. The crucial interaction parameters among DPD particles were evaluated by a series of calculations based on the fragment molecular orbital (FMO) method in a non-empirical way (Okuwaki *et al.*, *J. Phys. Chem. B*, 2018, **122**, 338–347). Through the DPD simulations, the water and hydrophilic particles aggregated, forming cluster networks surrounded by the hydrophobic phase. The structural features of formed water clusters were investigated in detail. Furthermore, the differences in percolation behaviors between Nafion and SPEEK revealed much better connectivity among water clusters by Nafion. The present FMO-DPD simulation results were in good agreement with available experimental data.

## Abstract 2

The mesoscopic structures of polymer electrolyte membrane (PEM) affect the performances of fuel cells.

## Graphical abstract

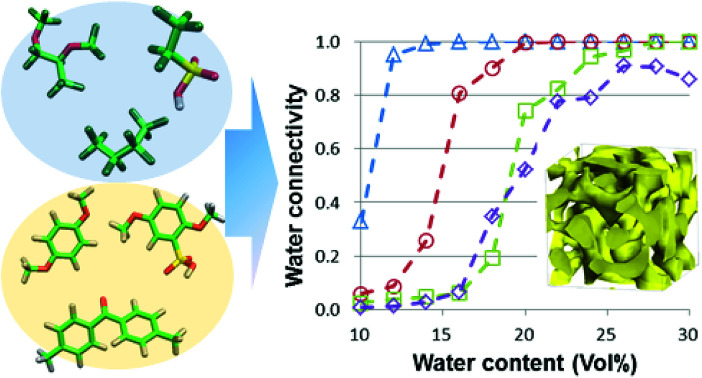

## 1. Introduction

Polymer electrolyte fuel cells (PEFC) – also called polymer exchange membrane fuel cell (PEMFC) – are attractive candidates for the use in vehicles because of no emission of carbon dioxide (CO_2_). The key component of PEFC is the polymer electrolyte membrane (PEM), and the most widely used PEM is of perfluorosulfonic acid (PFSA) type such as DuPont's Nafion®. In addition to its high proton conductivity, Nafion has chemical, thermal and mechanical stabilities.^1^ Nafion polymer consists of a polytetrafluoroethylene backbone (or Teflon®) and a side chain terminated with a sulfonic acid group. The structures formed by hydrated Nafion have been investigated by various experimental methods such as X-ray scattering,^2–6^ neutron scattering,^7–9^ transmission electron microscopy (TEM),^10^ atomic force microscopy (AFM),^11^ infrared spectroscopy (IR),^12^ and so on. As a result, it has been known that the hydrated Nafion membrane provides nanophase-segregated structures consisting of hydrophobic phase including main chains and hydrophilic phase containing a sulfonic acid group and water. Then, the formed water cluster-networks are related with the crucial proton conductivity.^2–16^ Radical reaction analysis using electron spin resonance has also been conducted.^17–19^

To elucidate the hydrated structure of Nafion, many molecular calculations have been carried out to date. Voth *et al.* analyzed the proton transport mechanism in Nafion at atomistic level using the self-consistent multistate empirical valence bond (SCMS-EVB) approach.^20–22^ Choe *et al.* simulated proton transport using the first principle molecular dynamics.^23–25^ Dupuis *et al.* performed a lot of molecular dynamics (MD) simulations for proton hopping and hydration of Nafion.^26–28^ Kawakami and Shigemoto also used MD simulation to verify the diffusion mechanism of proton.^29^ In addition, using density functional theory (DFT) calculation, water cluster interacting with sulfonic acid group,^30^ Nafion/Pt interface,^31^ and degradation of Nafion^32^ have been also reported.

Though Nafion has excellent conductivity as mentioned above, other types of PFSA which have different side chain length from Nafion have been studied.^33–35^ However, there is still room for improvement on chemical durability, gas permeability, high production cost, and so on. Alternatively, the sulfonated poly ether-ether-ketone (SPEEK) having aromatic hydrocarbon has attracted attention as a promising alternative to Nafion and related materials.^36,37^ SPEEK demonstrates high thermal stability, mechanical properties and high cost effectiveness, but the problems of chemical stability and poor conductivity have been pointed out.^38,39^ Against such a situation, Miyake *et al.* recently developed a new PEM composed of poly-phenylene with high chemical stability, and further improvements of the hydrocarbon PEM have been desired.^40^

In order to accelerate researches to optimize such material performances of PEM, theoretical studies based on molecular simulations (that deal with large sizes and time scales) should be useful. There are mainly two ways to perform the large-scale simulations as follows.

The first way is all-atom MD simulations using highly parallelized programs and huge computational resources. There have been various excellent programs such as MODYLAS,^41^ GROMACS,^42^ NAMD,^43^ and LAMMPS.^44^ In fact, extensive MD simulations for PEM have been reported as briefed below. Okazaki *et al.* investigated the morphology of the PEM using large-scale MD simulations.^45^ Knox and Voth also carried out atomistic MD simulations to probe morphological models of Nafion.^46^ Komarov *et al.* performed MD simulations with cell size of up to 36 nm including 4 million atoms.^47^

The second way is coarse-grained MD (CGMD) and related methods such as self-consistent mean field method (SC-MFT) and dissipative particle dynamics (DPD).^48–51^ These methods are very useful because large scale behaviors in molecular level are obtainable at reasonable computational costs. Many coarse-grained simulations have been performed for PEM. Voth *et al.* reported a mesoscale study of the proton transport using smoothed particle hydrodynamics (SPH).^52,53^ Wescott *et al.*^54^ and Galperin *et al.*^55^ used the polymer SC-MFT theory to reproduce the phase structure. Eikerling *et al.* performed a CGMD-based study of assembly of ionomer.^56,57^ DPD has been frequently used for researches of PEM. Morohoshi *et al.* used DPD to verify the structural dependences of physical properties of PEM^58,59^ and investigated the gas permeation by combining dynamic Monte Carlo (MC).^60^ Neimark *et al.* modeled the proton dissociation and conductivity.^61–63^Ref. 64–66 utilized DPD as well. Hereafter, we focus on DPD simulations.

The DPD method was proposed by Hoogerbrugge and Koelman,^66,67^ and was extended to polymer systems by Groot *et al.*^48–51^ However, the evaluation of a set of interaction parameters (*χ*) among particles have still been of difficult issue. There have been a couple of major routes of *χ* parameter predictions. The first route is based on the solubility parameter (SP) values. The SP value method was devised by Hildebrand,^68^ and various empirical estimation models such as atomic group contribution models^69–72^ were developed. Note that the SP values could be obtained from molecular simulations for single molecules as well.^71^ The second route is based on the evaluation of interactions between heterogeneous molecules, due to the contact energy between segments^72^ and the difference in cohesive energies of the aggregated model.^73^ This second route has advantage to directly determine the interactions between the contacting particles in a molecular level approach. Yamamoto and Hyodo performed novel DPD simulation using the parameter obtained from a classical force field (FF) molecular calculation and reproduced the network structure of the water clusters generated by Nafion.^74^ However, since the interaction between Nafion and water should involve both polarization and charge transfer, simulation results with FF might could have some limitations. For that reason, the parameters converted from experimental values were used for many of the simulations currently.

Certainly, simulations with non-empirical parameters are desirable to develop new type materials for which experimental data are hardly obtainable. In 2016, Sepehr *et al.* evaluated the effective interaction parameters of DPD for Nafion, based on *ab initio* molecular orbital (MO) calculations.^75^ Although *ab initio* evaluations of *χ* parameters for DPD are desirable, its applicability could be limited due to the enlarged computational costs when the molecular sizes of segment pairs grows. Therefore, we have developed a new approach^76,77^ to calculate such effective parameters based on the fragment molecular orbital (FMO) method.^78–82^ This procedure could be considered as an extension of Fan's method^72^ based on the Flory–Huggins theory.^83,84^

In this paper, we report a series of DPD-based investigations on the morphology of hydrated Nafion and SPEEK. The crucial interaction parameters among DPD particles were evaluated through our new protocol with FMO.^76,77^ Furthermore, the network connectivity of water clusters was evaluated through the percolation analysis of formed water clusters in PEM,^15,58,85–88^ since such an effective index should relate with the conductivity. Nafion and SPEEK were compared. The rest of this paper is configured as follows. In Section 2, the *χ* parameter evaluations and models used for DPD simulations are described in detail. In Section 3 the simulated results are presented and discussed.

## 2. Simulation details

### 2.1. *χ* Parameter evaluation

In the Flory–Huggins lattice theory, the free energy change (Δ*G*) for a binary system is expressed as^83,84^1
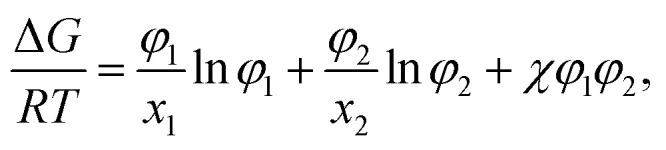
where *φ*_*i*_ and *x*_*i*_ are the volume fraction and the chain length (*i* = 1, 2 for the two components), respectively. The first and the second terms on the right side of this equation describe the entropy changes, and the third term provides the enthalpy change. The *χ* parameter is defined as2
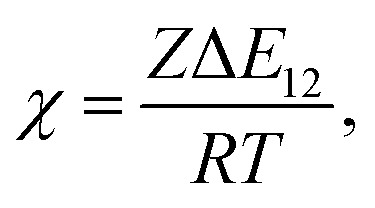
where *Z* is the coordination number of the model lattice and the contact energy Δ*E*_12_ is given by the following equation,3
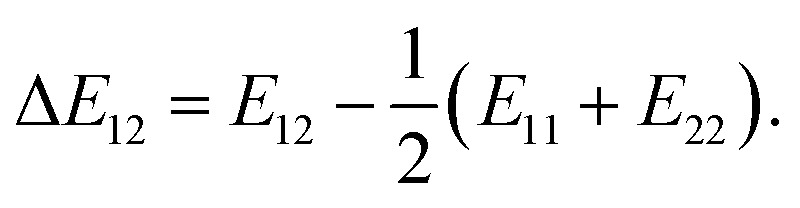
*E*_*ij*_ in this equation is the average interaction energy between the segments *i* and *j* in the two components, and Δ*E*_12_ corresponds to the energy gain per segment due to the mixing. These relations imply a scale down of problem from mesoscale to nanoscale. Fan *et al.* proposed the procedure to calculate *Z* and Δ*E*_12_, based on MC simulations with classical FF set.^72^

Recently, we have developed a new procedure^76,77^ to estimate the *χ* parameter set through a series of FMO calculations,^80,81^ where the electronic effects of polarization and charge transfer were incorporated in the energy evaluation and also the molecular anisotropy was taken into account. Consequently, the *χ* parameter could be calculated using the following equation4
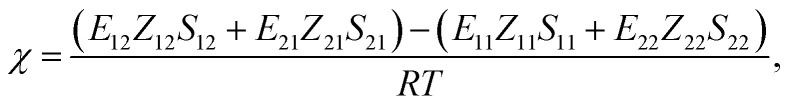
where *Z*_*ij*_ is the coordination number of segment *j* around segment *i*, and *S*_*ij*_ corresponds to the scaling factor associating with anisotropy (refer to ref. 78 and 79 for details).

**Fig. 1:**
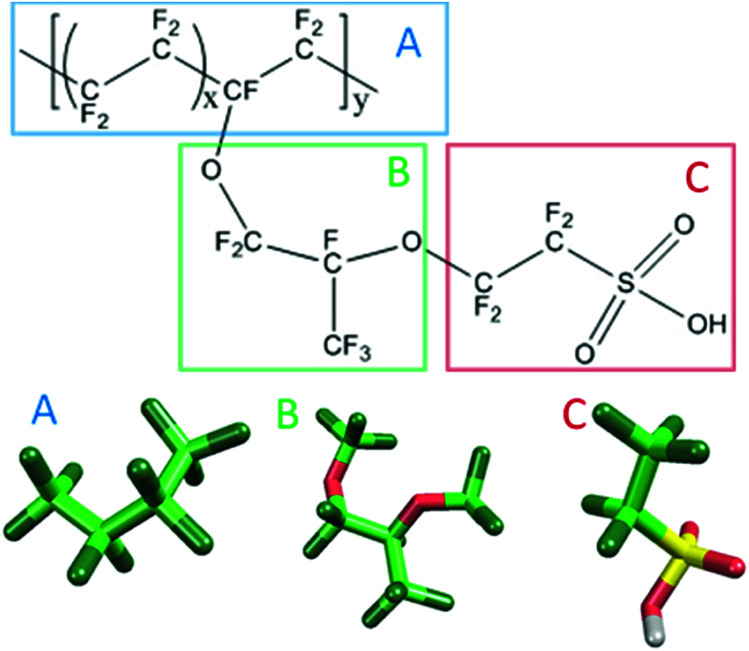
Molecular structure and segment models of Nafion monomer.

**Fig. 2:**
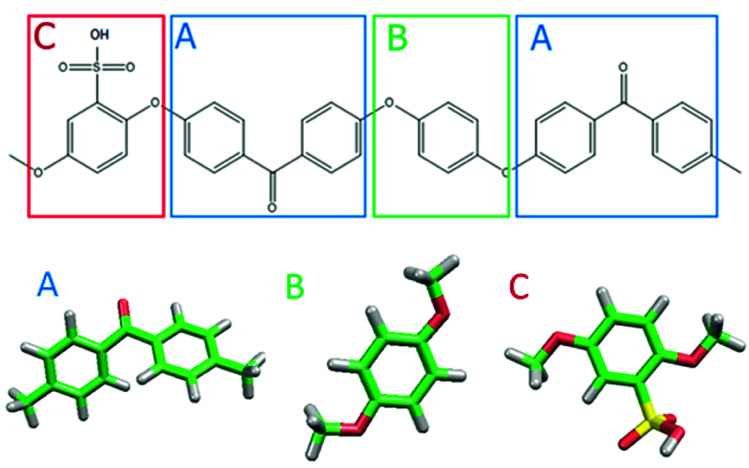
Molecular structure and segment models of SPEEK monomer.

**Fig. 3:**
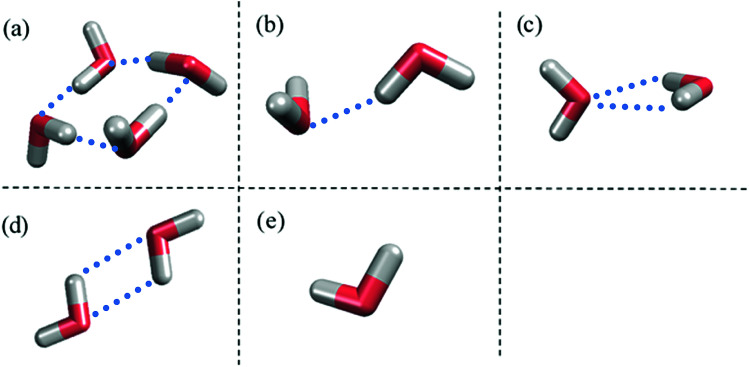
Various water structures used for parameter evaluations. (a) Tetramer. (b) Linear dimer. (c) Bifurcated dimer. (d) Cyclic dimer. (e) Monomer. Blue dots indicate hydrogen bonds between water molecules.

Fig. 1 shows the structure of Nafion. According to Yamamoto's previous study,^74^ the basic unit of Nafion chain is divided into three segments of the same size (A: –CF_2_–CF_2_–CF_2_–CF_2_–, B: –O–CF_2_–C(CF_3_)FO–, and C: –CF_2_–CF_2_–SO_3_H). The termini were capped with F for segments A and C and with CF_3_ for segment B. The structure of SPEEK was shown in Fig. 2. The chain was divided into three segments A, B, and C similarly to Nafion. The termini were capped with CH_3_. Furthermore, various conformations were considered for water molecules. The water particle (W) is typically modeled by a water tetramer with cyclic hydrogen bonding for DPD simulations due to the segment size problem, as was done in ref. 74. However, such a model might fail to interact with outer particles because of its internal hydrogen bonding. Therefore, we employed three kinds of dimers (with shapes of linear, cyclic, and bifurcated types) and even a monomer as the candidates interacting with sulfonic side chain (C). All the water structures employed in the parameter evaluation are shown in Fig. 3.

The geometries of segments were optimized at the dispersion-corrected B97D (ref. 89) DFT level with the 6-31G(d′,p′) basis set^90,91^ by using the GAUSSIAN09 program.^92^ Note that the orbital exponents of polarization functions of 6-31G(d′,p′) were optimized for the respective elements. The generation of geometrical configurations (each number was typically 2000) was performed with the J-OCTA program.^93^ A number of FMO calculations for all the possible combinations among segments were carried out at FMO-MP2/6-31G(d′) level, where the ABINIT-MP program^79^ was used in parallel executions on several in-house servers with Intel's Xeon processors.

### 2.2. DPD simulation

DPD is a sort of soft particle dynamics with conservative, dissipative and random forces.^66,67^ The fundamental DPD scheme was extended to polymer systems by Groot *et al.* by introducing a bead-spring type particle model.^48–50^ The outline of Groot's DPD model is described below. The time evolution of the given system is simulated by solving the Newton equation of motion5
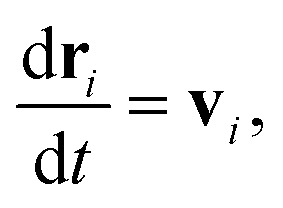
and6
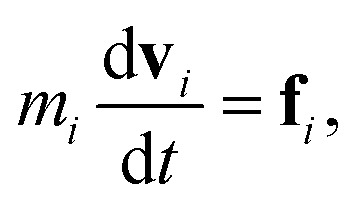
where, **r**_*i*_, **v**_*i*_, and *m*_*i*_ are the position, velocity, and mass of the particle *i*, respectively. For convenience, the masses and diameter of the particles are scaled relative to 1. The force **f**_*i*_ contains four parts as7
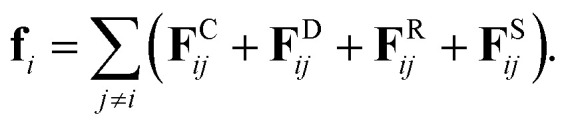
The first three forces of the right-hand-side are considered within a certain cutoff radius *r*_c_. The conservative force **F**^C^_*ij*_ is a soft repulsion action as follows8
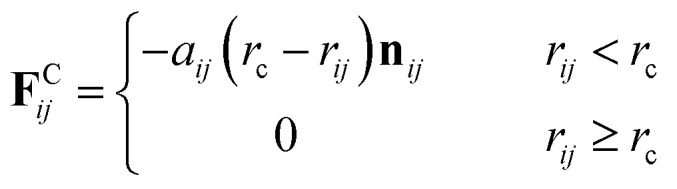
where *a*_*ij*_ is the maximum repulsion force between particles *i* and *j*, and related definitions are **r**_*ij*_ = **r**_*j*_ − **r**_*i*_, **r**_*ij*_ = |**r**_*ij*_|, and **n**_*ij*_ = **r**_*ij*_/|**r**_*ij*_|. The repulsion parameters between different type particles correspond to the mutual solubility provided by the *χ* parameter set. When the reduced density *ρ* is assumed to be 3, a linear relation with *χ*_*ij*_ is usually set as^48,49^

9 *a*_*ij*_ = *a*_*ii*_ + 3.27*χ*_*ij*_,

In eqn (7), the dissipative force **F**^D^_*ij*_ represents the hydrodynamic drags, and the random force **F**^R^_*ij*_ incorporates thermal noises of the Gaussian statistics. The fourth force **F**^S^_*ij*_ is a harmonic force calculated for particles directly connected with spring bonds. The detailed functional forms of these potentials are described in eqn (S1)–(S8) in the ESI,† and the numerical values of associated parameters are compiled in Tables S1 and S2.†

**Fig. 4:**
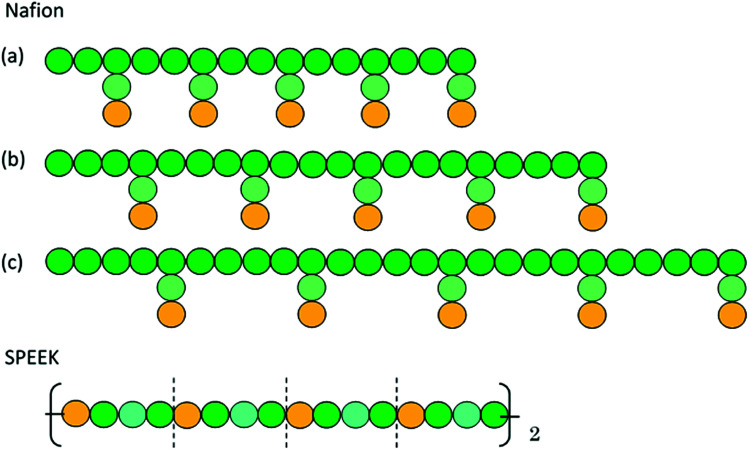
DPD particle models for Nafion polymer and SPEEK. Particle size is approximately 0.71 nm. Particles with Green, light green and yellow colors indicate segments A, B and C, respectively, shown in Fig. 1 for Nafion and in Fig. 2 for SPEEK. Symbolic structure (b) corresponds to Nafion 117 (EW = 1100),^33^ and structures (a) and (c) are defined as low and high EW models, respectively.

In the present study, the time evolution was calculated by the modified Verlet algorithm^48^ with the empirical factor *λ* = 0.65 and time step Δ*t* = 0.05. The cubic cell system was defined as a dimension of *L* = 30 DPD length unit, corresponding to 23.5 nm in real unit (DPD particle diameter is 0.71 nm).^51^ Each simulation involved about 81 000 beads, and the beads were initially packed randomly at the standard mean density *ρ* = 3.^48^ The cut-off radius *r*_c_ was set to 1. A default value of 4 (ref. 51) was used for the spring constant of **F**^S^_*ij*_. The polymer models used are shown in Fig. 4. Three types of symbolic structures which have different equivalent weight (EW) were prepared for Nafion. In Fig. 4, the structure (b) corresponds to Nafion 117 (EW = 1100).^33^ For SPEEK, one hydrophilic segment was placed for every four particles. The time integration was performed for 10 000 steps corresponding to 500 DPD-time unit (*t*). The simulations were carried out for water contents of 10–30 vol% with 2 vol% intervals. The trajectory data were saved every 100 steps (5 DPD-time unit) for subsequent analyses. All the DPD simulations were performed with the COGNAC program^93,94^ on in-house servers.

## 3. Results and discussion

### 3.1. Results of the parameter evaluation

*E*_*ij*_ and *S*_*ij*_ between various water models and hydrophilic segment C are shown in Table 1. It is found that the *E*_*ij*_ value varies greatly depending on the water structures. The interaction energy with the linear dimer is the strongest one, as expected from its open shape for external interactions. This linear shape of water dimer was thus actually used in evaluating the interaction energies with Nafion, where the coordination number in eqn (2) was still evaluated with the tetramer. The *E*_*ij*_, *S*_*ij*_ and *Z*_*ij*_ between each segment pair at 350 K of Nafion are shown in Table 2. The *E*_*ij*_ values among the hydrophobic segments (A–A, A–B, B–B) are approximately −1 kcal mol^−1^, whereas the interaction energies between the hydrophilic segments (C–C, C–W, W–W) are about −10 kcal mol^−1^. On the other hand, the *S*_*ij*_ values between hydrophobic segments are about 0.9, and those between hydrophilic segments are less than or equal to 0.5. These facts imply that hydrophobic segments are isotropic but hydrophilic segments are anisotropic. Table 3 shows the values of *χ* obtained from the results listed in Table 2 using eqn (4), where the values of Yamamoto and Hyodo's preceding study^74^ are listed for comparison. The *χ* value using FMO is small for A–B (−0.17) and C–W (−4.1) pairs, while it exceeds 20 for A–W and B–W pair. Although the tendency of these parameters is consistent with the results of ref. 74, the absolute values of our *χ* related to water particles is larger, suggesting the contributions of polarization and charge transfer interactions incorporated by the FMO calculations.

**Table 1:** Interaction energies (*E*_*ij*_), and scaling factors (*S*_*ij*_) of segment C of Nafion shown in Fig. 1 and various water models (W) at 350 K

	Tetramer	Dimer			Monomer
		Bifurcated	Cyclic	Linear	
*E*_*ij*_	−10.18	−12.93	−10.88	−15.58	−10.33
*S*_12_ (C–W)	0.24	0.23	0.25	0.20	0.20
*S*_21_ (W–C)	0.45	0.27	0.44	0.30	0.52

**Table 2:** Interaction energies (*E*_*ij*_), scaling factors (*S*_*ij*_), and coordination numbers (*Z*_*ij*_) for each segment of Nafion and water at 350 K. Note that label W corresponds to linear shape of water dimer for C–W pair and tetramer for the others

		Segment 1											
		A			B			C			W		
		*E*_*ij*_	*S*_*ij*_	*Z*_*ij*_	*E*_*ij*_	*S*_*ij*_	*Z*_*ij*_	*E*_*ij*_	*S*_*ij*_	*Z*_*ij*_	*E*_*ij*_	*S*_*ij*_	*Z*_*ij*_
Seg. 2	A	−0.76	0.89	7.2	−0.87	0.91	8.0	−0.82	0.83	8.1	−1.32	0.84	8.1
	B	−0.87	0.90	7.0	−0.95	0.89	8.0	−0.91	0.84	8.2	−1.38	0.83	7.3
	C	−0.82	0.92	7.6	−0.91	0.90	8.7	−4.26	0.46	8.6	−15.58	0.30	7.4
	W	−1.32	0.83	13.7	−1.38	0.83	12.3	−15.58	0.20	14.2	−9.87	0.51	10.6

**Table 3:** Calculated *χ* parameters and corresponding repulsion parameters *a*_*ij*_ for each pair of hydrated Nafion

	This work		Previous work^74^
	*χ*	*a*_*ij*_	*χ*
A–B	−0.17	24.44	0.02
A–C	7.51	49.54	3.11
B–C	7.36	49.05	1.37
A–W	25.37	107.91	5.79
B–W	27.86	116.05	4.90
C–W	−4.1	11.60	−2.79

The energy values with the water dimer of linear form were used for parameter evaluation for SPEEK as well as Nafion. *E*_*ij*_, *S*_*ij*_ and *Z*_*ij*_ between each segment pair at 350 K of SPEEK are shown in Table 4. The interactions between hydrophobic particles are about 1.5 times larger than that of Nafion. The dispersion interaction caused by the π electron of benzene rings may be responsible for this enhancement. In addition, segment C having sulfonic acid has a strong interaction with other main chain segments, and also interacts with water particles greatly. The value of *χ* obtained from the above results is shown in Table 5. The *χ* values among polymer segments (A–B, A–C, and B–C) were −0.75, 4.94, and 3.53 respectively, suggesting high affinity for each other. In addition, the *χ* of C–W is −3.78, which indicates strong interaction. Thus, the tendency of the parameters predicted from the structure of SPEEK is reproduced. The final *a*_*ij*_ values of Nafion and SPEEK for DPD are found in Table S2.†

**Table 4:** Interaction energies (*E*_*ij*_), scaling factors (*S*_*ij*_), and coordination numbers (*Z*_*ij*_) for each segment of SPEEK and water at 350 K. Note that label W corresponds to linear shape of water dimer for C–W pair and tetramer for the others

		Segment 1											
		A			B			C			W		
		*E*_*ij*_	*S*_*ij*_	*Z*_*ij*_	*E*_*ij*_	*S*_*ij*_	*Z*_*ij*_	*E*_*ij*_	*S*_*ij*_	*Z*_*ij*_	*E*_*ij*_	*S*_*ij*_	*Z*_*ij*_
Seg. 2	A	−1.27	0.82	8.2	−1.27	0.89	11.0	−2.38	0.76	9.6	−0.98	0.88	9.2
	B	−1.27	0.85	7.1	−1.28	0.89	9.2	−2.28	0.85	7.9	−1.25	0.87	8.3
	C	−2.38	0.77	7.5	−2.28	0.78	11.0	−6.38	0.55	8.3	−8.13	0.76	8.2
	W	−0.98	0.84	17.6	−1.25	0.88	24.5	−8.13	0.37	18.1	−9.89	0.50	10.6

**Table 5:** Calculated *χ* parameters and corresponding repulsion parameters *a*_*ij*_ for each pair of hydrated SPEEK

Pair	*χ*	*a*_*ij*_
A–B	−0.75	22.55
A–C	4.94	41.14
B–C	3.53	36.54
A–W	28.09	116.80
B–W	19.49	88.69
C–W	−3.78	12.65

### 3.2. Results of DPD simulation

**Fig. 5:**
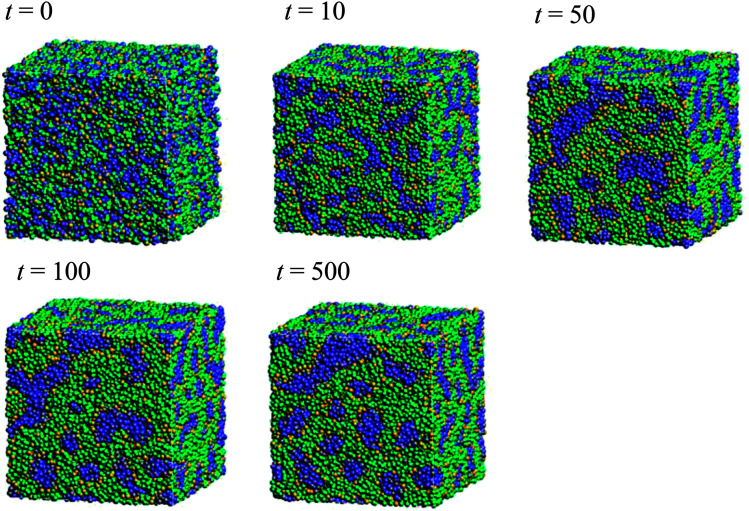
Time-dependent (with symbol “*t*”) morphologies of the case of 20 vol% water content for Nafion with symbolic structure (b) shown in Fig. 4. Hydrophobic, hydrophilic, and water particles are depicted with green, yellow and blue colors, respectively.

**Fig. 6:**
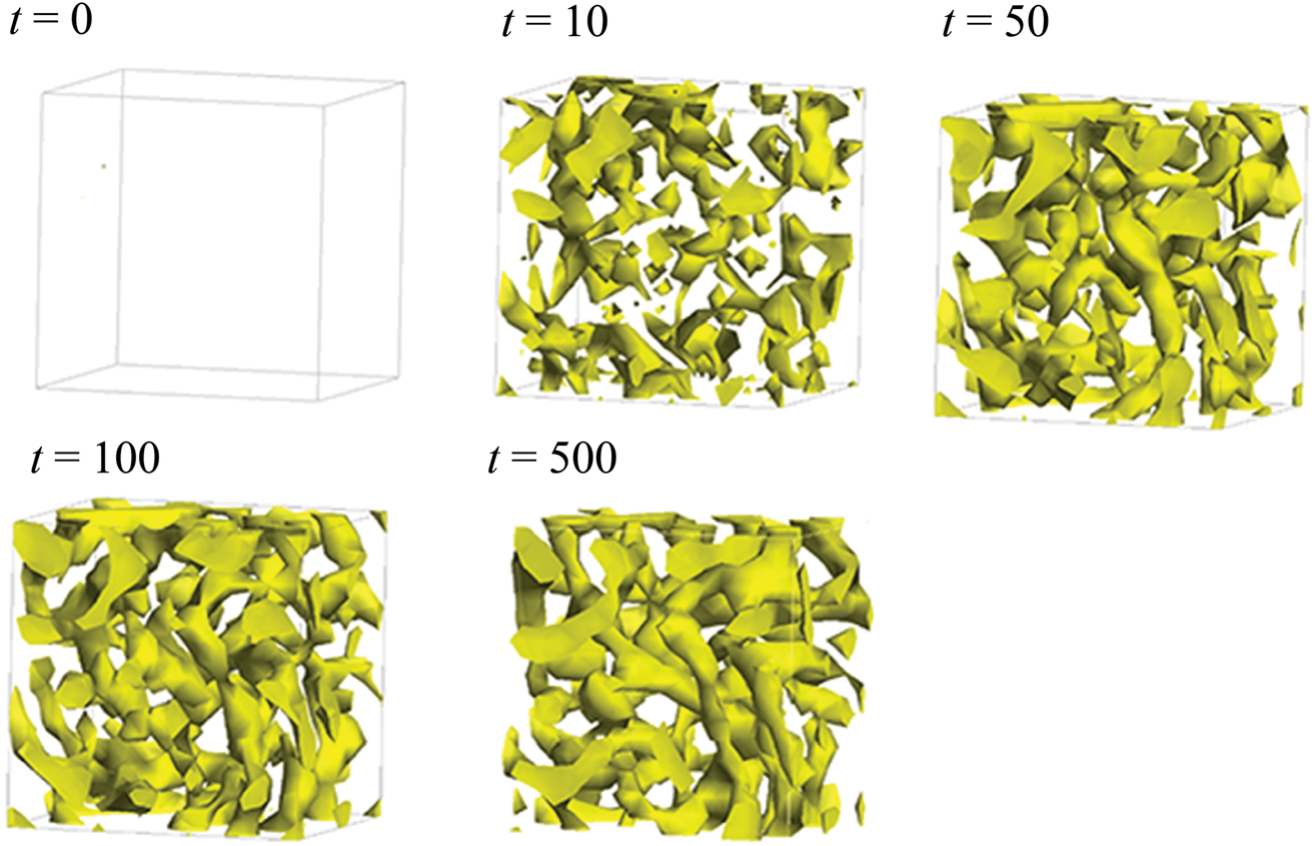
Time-dependent water density distributions of the case of 20 vol% water content for Nafion with symbolic structure (b) shown in Fig. 4. The regions in which more than 40% of water particles exist are displayed with yellow color.

Fig. 5 illustrates the time-dependent morphologies of the 20 vol% hydrated Nafion with symbolic structure (b) of Fig. 4. It is visible that small domains of water aggregation develop into large water clusters surrounded by hydrophilic particle of Nafion. Fig. 6 shows the time-dependent water density distribution (for more than 40% of water particles) of the same simulation case. The complicated network structures of water clusters are being formed from the initial uniform situation with respect to the time evolution. This progress is consistent with the visualized results of Fig. 5.

**Fig. 7:**
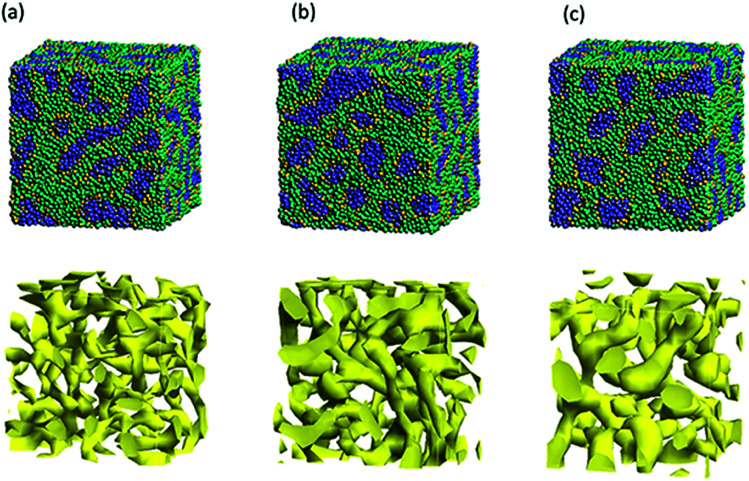
Structural changes due to the differences in EW of the case of 20 vol% water content for Nafion. Labels (a), (b) and (c) correspond to symbolic structures shown in Fig. 5. Upper row shows morphologies, and lower rows does water density distributions.

**Fig. 8:**
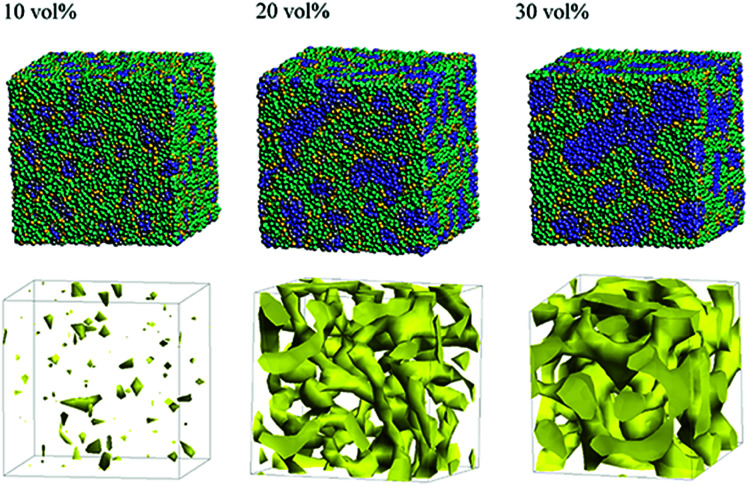
Dependence of morphology on water contents for Nafion (*t* = 500) with symbolic structure (b) in Fig. 4. Morphologies of 10, 20, and 30 vol% are depicted in upper row. Water density distributions are given drawn in lower row.

**Fig. 9:**
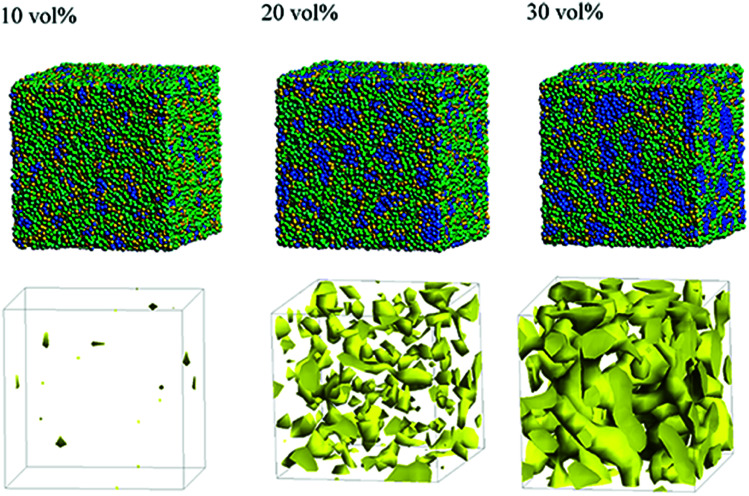
Dependence of morphology on water contents for SPEEK (*t* = 500). Morphologies of 10, 20, and 30 vol% are depicted in upper row. Water density distributions are given drawn in lower row.

Next, the structural changes due to the differences in EW of Nafion (corresponding to the symbolic structures of (a), (b) and (c) in Fig. 4) are investigated. The results of 20 vol% case are shown in Fig. 7. From this figure, one can see the behavior that the size of water cluster (lower row) enlarges as the chain (or length of segment A of Nafion) elongates. Fig. 8 illustrates the dependence of morphology on water contents of 10, 20 and 30 vol% for the Nafion, and Fig. 9 does the corresponding results for SPEEK. Comparison between Fig. 8 and 9 indicate that the water clusters formed by Nafion connect each other more easily than those formed by SPEEK. Especially, the water clusters look still rather segmented at 20 vol% for SPEEK, although the mutual connections almost complete for Nafion. A probable reason for this difference is the fact that there are more potential sites for hydrogen bond with water in SPEEK than in Nafion. In short, Nafion could provide better connectivity of water clusters than does SPEEK.^86^

### 3.3. Analysis of Nafion cluster structure

**Fig. 10:**
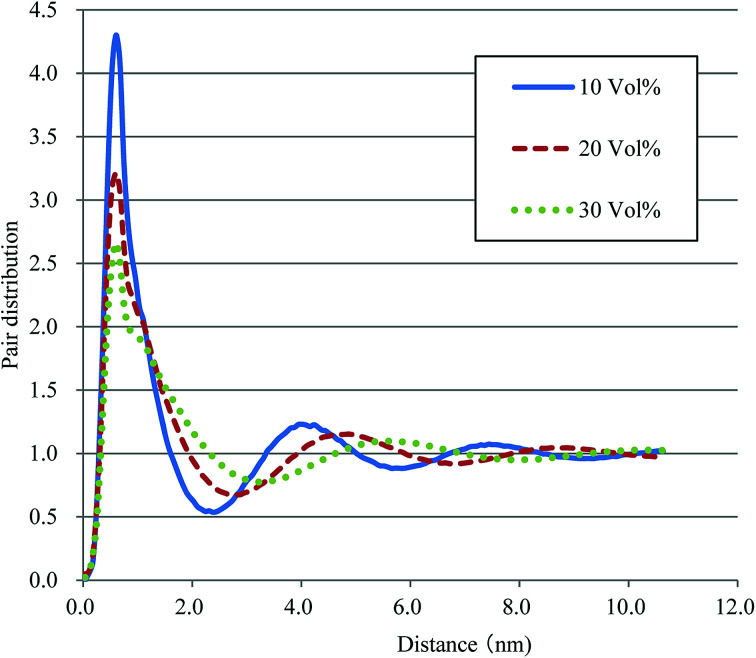
Radial distribution functions of water particles for Nafion (*t* = 500). Solid, dashed and dotted lines plot the results of 10, 20, and 30 vol% water contents, respectively.

**Fig. 11:**
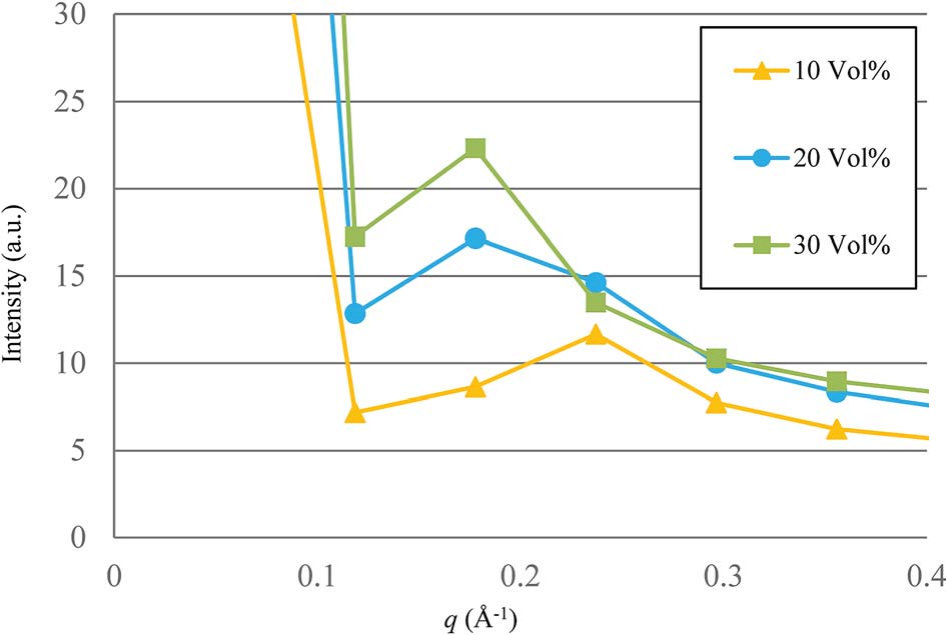
Calculated small-angle scattering patterns for Nafion (*t* = 500). Symbols of triangle, circle and square represent the results of 10, 20 and 30 vol% water contents, respectively.

Based on the qualitative discussion on the mesoscopic structures of water clusters in the previous section, comparative discussion with available experimental data is made for Nafion. Fig. 10 illustrates the radial distribution functions (RDFs) of water particles for Nafion with symbolic structure (b) (Nafion 117), where the three cases of water contents of 10, 20 and 30 vol% are plotted. The Fourier transformation of RDF could provide the small-angle scattering pattern,^74^ as plotted in Fig. 11. The peak derived from the water-network (or so-called ionomer peak) is observed around *q* = 0.2. When the water content increments, this peak shifts to lower angles and its intensity increases. This tendency was in good agreement with the experimental data of small angle neutron scattering (SANS) (refer to Fig. 6 of ref. 8).

**Fig. 12:**
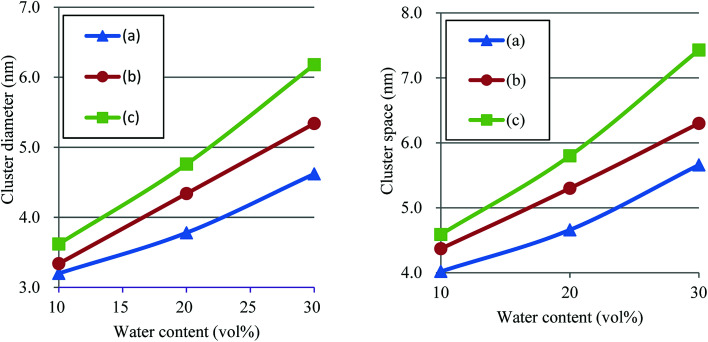
Dependences of cluster diameter and space on water contents of 10, 20 and 30 vol% for Nafion. Symbols of triangle, circle and square represent the results of symbolic structures (a), (b) and (c) in Fig. 4.

According to the precedent study^74^ by Yamamoto and Hyodo, the diameter of water cluster was measured at the point where *g*(*r*) became 1 after the first peak (see again Fig. 10). The separation between the first and second peaks was taken as the cluster space. Fig. 12 plots the dependences of cluster diameter and space on water contents of 10, 20 and 30 vol%. The incremental trend is observed for both diameter and space against the water content. For Nafion 117 (symbolic structure (b) in Fig. 4), as the water content increases for 10–30 vol%, the cluster diameter changes to 3.3–5.3 nm and its spacing changes to 4.4–6.3 nm. The corresponding experimental values of the cluster diameter and distance are 4 nm and 5 nm, respectively,^2,16^ being in good agreement with the present simulation results. The elongation of chain length (or the decreasing of EW) leads to the increase of both cluster size and space, and this trend is consistent with the result reported by Morohoshi *et al.*^60^

### 3.4. Evaluation of diffusion coefficient

Diffusions of protons in the mesoscopic water network has been considered as a crucial indicator of performance for the PEM.^95^ The diffusion coefficients (*D*) were thus evaluated from the DPD results, based on the mean square displacement (MSD) of *t* = 150–450 by using the following relation

10 〈(*r*(*t*) − *r*(0))^2^〉 = 6*Dt*.

**Fig. 13:**
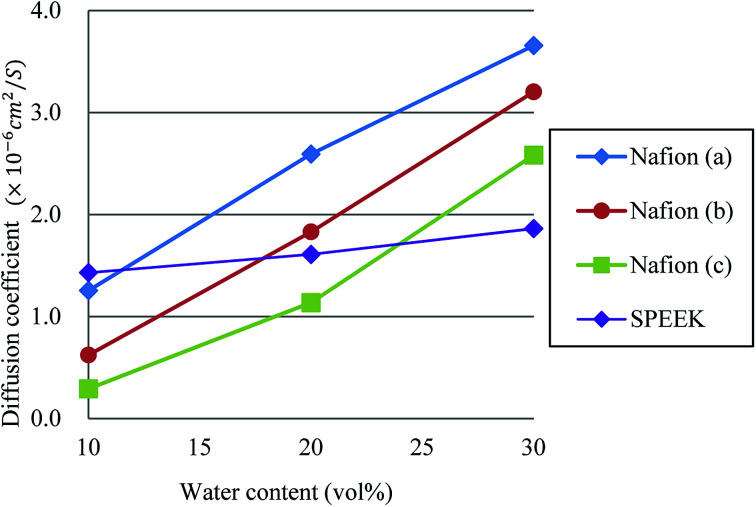
Dependences of diffusion coefficient on water contents for Nafion and SPEEK. Symbols of triangle, circle, and square represent the results of symbolic structures (a), (b), and (c) of Nafion in Fig. 4, respectively. Symbol of diamond corresponds to SPEEK.

Fig. 13 plots the dependences of diffusion coefficient on water contents for Nafion and SPEEK. For Nafion, all three cases show almost linear relation against the water content. Although a linear relation is found for SPEEK as well, its slope is rather small, reflecting the difference in connectivity of water clusters from Nafion. In other words, Nafion should provide better connections for proton diffusions. The hydration level *λ*, which is the number of water molecules per sulfonate part, for water contents of 10, 20 and 30 vol% are 2.5, 6 and 10, respectively, for Nafion 117 (structure (b)). According to experiments of Zawodzinski *et al.*,^95^ the diffusion coefficients of that region are roughly 0.8 × 10^−6^ to 4.5 × 10^−6^ cm^2^ s^−1^. The present DPD-based results have a corresponding range of 0.6 × 10^−6^ to 3.2 × 10^−6^ cm^2^ s^−1^, providing reasonable agreement with the experimental data.

### 3.5. Percolation analysis

So far, several differences due to the connectivity of water clusters between Nafion and SPEEK have been discussed. To directly evaluate the connectivity value, the percolation analysis was performed. The size of water cluster was defined as

**Fig. 14:**
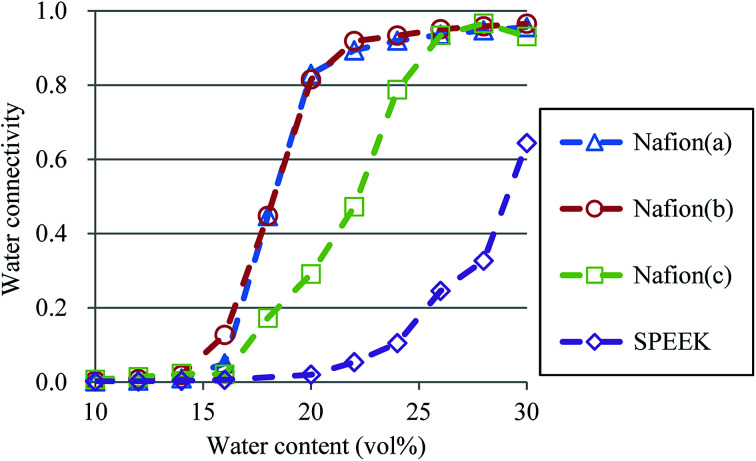
Water connectivity plots for Nafion and SPEEK with respect to water contents using the transient structure of every 100 steps. Symbols of triangle, circle, and square represent the results of symbolic structures (a), (b), and (c) of Nafion in Fig. 4, respectively. Symbol of diamond corresponds to SPEEK.

**Fig. 15:**
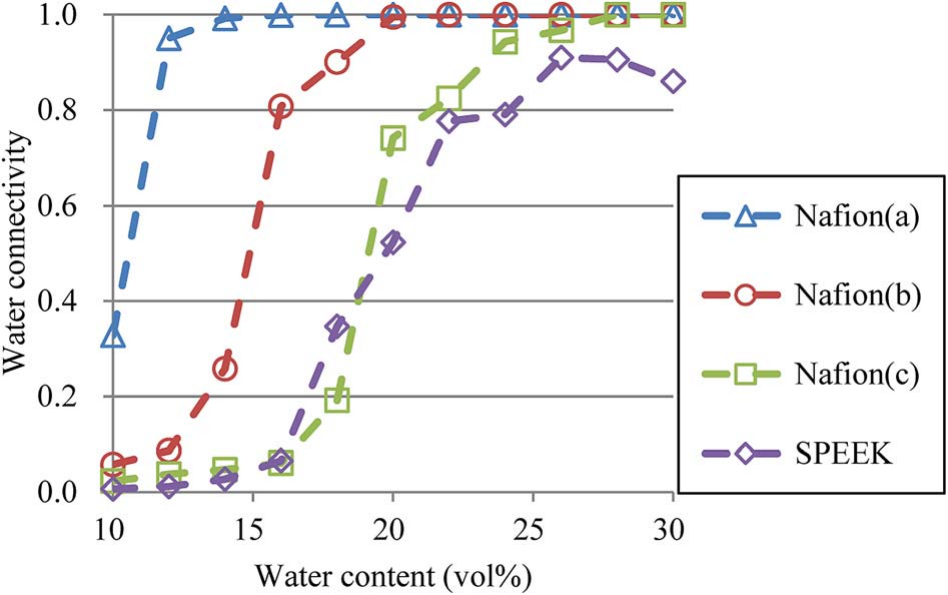
Water connectivity plots for Nafion and SPEEK with respect to water contents using the average structure of every 100 steps. Symbols of triangle, circle, and square represent the results of symbolic structures (a), (b), and (c) of Nafion in Fig. 4, respectively. Symbol of diamond corresponds to SPEEK.

11 *R*(*i*,*j*) ≤ *R*_C_,

where *R*(*i*,*j*) is the distance between particle *i* and *j*, and *R*_C_ is a criterion for contact. If eqn (11) is true, these two particles (*i* and *j*) belong to the same cluster. The cluster connectivity *M* is thus calculated as follows12
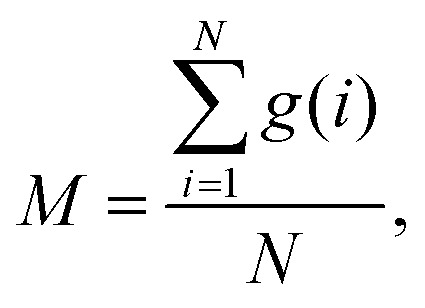
where *N* is the total number of particles in the system, and *g*(*i*) is the cluster size to which particle *i* belongs. The *R*_C_ was set to 1.1 DPD unit length, corresponding to the spacing of the first coordination area obtained from the RDF of DPD (see Fig. 10). For the percolation analysis, a series of additional DPD simulations were carried out for the water content range of 10–30 vol% with 2 vol% intervals. Fig. 14 presents the results of connectivity for Nafion and SPEEK, where the transient structures of every 100 steps of *t* = 300–500 were used in the evaluations. It is found that the connectivity rapidly grows over 0.8 at about 20 vol% for Nafion with the symbolic structures of (a) and (b) and that there is a delay in grow for the symbolic structure (c). Finally, the connectivity value reach almost one (as full connection) as expected. These critical behaviors qualitatively accord with an experimental threshold of 10 vol%. In addition, for a given value of *λ* (or hydration level), the connectivity became slightly higher with an increase in EW. This tendency was in good agreement with Fontanella's experimental observation.^88^ On the other hand, the critical grow of connectivity occurs at about 30 vol% for SPEEK, and this tendency agrees with the experimental result by Wu *et al.*^86^ Since the connection structure of water clusters should be different between Nafion and SPEEK, an alternative scheme was tried for the percolation analysis where the structures were averaged in every 100 steps and the *R*_C_ value was loosed to 0.8 DPD unit (the first peak position of the RDF). The corresponding results are plotted in Fig. 15. For Nafion, the plots of connectivity shift toward lower water content, and a degeneracy of (a) and (b) is resolved. The correspondence to the experimental results^88^ is improved by a certain extent. The results for SPEEK are lower shifted, but the final connectivity is still about 0.9 (see also Fig. S1† of time-dependent connectivity when necessary). Both relative positioning of hydrophilic/hydrophobic beads and length of polymer chains should affect the connectivity, as seen in the situation that the results of Nafion (c) model having long chain are rather close to those of SPEEK model in Fig. 15. In summary, the percolation analysis has shed on light on the difference in mesoscale connection structures of water clusters formed by Nafion and SPEEK.^86^ Certainly, Nafion is better as the PEM material than SPEEK.

## 4. Conclusion

In the present study, we investigated the mesoscopic structures of Nafion and SPEEK by using DPD simulations. A crucial set of effective interaction (*χ*) parameters has been determined with a series of non-empirical FMO calculations for all the possible particle–particle interactions.^76^ The results of DPD simulations were analyzed in several ways, illuminating the differences in mesoscopic structures of water clusters between Nafion and SPEEK. The simulation data were compared with available experimental data, and reasonable agreement have been obtained, indicating the reliability of non-empirical FMO-DPD scheme. Our analysis showed that the connectivity of water clusters formed by Nafion is better that by SPEEK. This difference could be considered as a reason why Nafion has been the first choice of PEM materials industrially. Our simulation results have been in accord with other studies.^45,74,86,88^ We would believe that the present FMO-DPD simulation scheme has a wide-range applicability to various mesoscopic systems. Several demonstrative applications such as lipid-water system^96–98^ have been reported. Deng *et al.* have made a DPD study of sulfonic gemini surfactants.^99^ Our present approach may be applicable to such novel materials. Very recently, Inokuchi *et al.* published an interesting paper in which DPD results of surfactants were analyzed and predicted by machine learning (ML) techniques.^100^ The combination of DPD and ML should be suggestive for our future works. Finally, we would note that the sequenced protocols to evaluate a set of *χ* parameters through the FMO calculations has been packaged as a workflow system (FCEWS, FMO-based Chi-parameter Evaluation System).^77^

## Conflicts of interest

The authors declare no competing financial interest.

## Supplementary Material

RA-008-C8RA07428C-s001
