# Supplementary material for: Theoretical analyses on water cluster structures in polymer electrolyte membrane by using dissipative particle dynamics simulations with fragment molecular orbital based effective parameters
Source: RSC Adv. 2018 Oct 8;8(60):34582–95. doi: 10.1039/c8ra07428c (PMC9086946; doi:10.1039/c8ra07428c)

Electronic Supplementary Material (ESI) for RSC Advances.
This journal is © The Royal Society of Chemistry 2018

Supporting Information

Theoretical Analyses on Water Cluster Structures in Polymer Electrolyte Membrane by Using Dissipative Particle Dynamics Simulations with Fragment Molecular Orbital Based Effective Parameters

Koji Okuwaki[1], Yuji Mochizuki[1,2*], Hideo Doi[1$], Shutaro Kawada[1], Taku Ozawa[3], Kenji Yasuoka[4]

[1] Department of Chemistry and Research Center for Smart Molecules, Faculty of Science, Rikkyo University, 3-34-1 Nishi-ikebukuro, Toshima-ku, Tokyo 171-8, Japan

[2] Institute of Industrial Science, The University of Tokyo, 4-6-1 Komaba, Meguro-ku, Tokyo 153-8505, Japan

[3] JSOL Corporation, 2-5-24 Harumi, Chuo-ku, Tokyo 104-0053, Japan

[4] Department of Mechanical Engineering, Keio University, Yokohama 223-8522, Japan

[$] Present address: Research Center for Computational Design of Advanced Functional Materials, National Institute of Advanced Industrial Science and Technology (AIST), Central 2, 1-1-1 Umezono, Tsukuba-shi, Ibaraki 305-8568, Japan

(*email for correspondences: fullmoon@rikkyo.ac.jp)

Functional forms of the potentials used in DPD

In Eq. (7) of the main text, the dissipative force $\mathbf{F}_{ij}^{D}$ is a hydrodynamic drag and is given by[1]

$$\mathbf{F}_{ij}^{D} = \begin{cases} -\gamma\omega^{D}(r_{ij})(\mathbf{n}_{ij}\cdot\mathbf{v}_{ij})\mathbf{n}_{ij} & r_{ij} < r_c \\ 0 & r_{ij} \geq r_c \end{cases}, \quad \text{(S1)}$$

where γ is a friction parameter related to the viscosity of the given system. $\omega^{D}(r_{ij})$ is the weighting function and the velocity difference is defined as $\mathbf{v}_{ij} = \mathbf{v}_j - \mathbf{v}_i$.

The random force $\mathbf{F}_{ij}^{R}$ corresponds to thermal noise and is governed by the parameter σ and another weighting function $\omega^{R}(r_{ij})$ as follows:[1]

$$\mathbf{F}_{ij}^{R} = \begin{cases} \sigma\omega^{R}(r_{ij})\zeta_{ij}\Delta t^{-1/2}\mathbf{n}_{ij} & r_{ij} < r_c \\ 0 & r_{ij} \geq r_c \end{cases}. \quad \text{(S2)}$$

The randomness is incorporated through the element ζ_{ij}, which is a randomly fluctuating variable with Gaussian statistics,

$$\langle\zeta_{ij}(t)\rangle = 0, \quad \text{(S3)}$$

$$\langle\zeta_{ij}(t)\zeta_{kl}(t')\rangle = (\delta_{ik}\delta_{jl} + \delta_{il}\delta_{jk})\delta(t - t') . \quad \text{(S4)}$$

They are assumed to be uncorrelated for different particle pairs and time. There is a relation between two weighting functions and two parameters,

$$\omega^{D}(r_{ij}) = [\omega^{R}(r_{ij})]^2 \quad \text{(S5)}$$

$$\sigma^2 = 2\gamma kT . \quad \text{(S6)}$$

In our simulation, the weighting function was chosen as follows:[1]

$$\omega^{D}(r_{ij}) = [\omega^{R}(r_{ij})]^2 = \begin{cases} (r_c - r_{ij})^2 & r_{ij} < r_c \\ 0 & r_{ij} \geq r_c \end{cases}. \quad \text{(S7)}$$

The variable $\Delta t^{-1/2}$ in Eq. (S2) is used to ensure the consistent diffusion of particles independent of the step size of the integrations.[1,2]

The spring force $\mathbf{F}_{ij}^{S}$ for a polymer is considered as harmonic springs for the equilibrium bond distance $\boldsymbol{r}_s$ if i is connected to j,[1]

$$\mathbf{F}_{ij}^{S} = -C(\boldsymbol{r}_s - \boldsymbol{r}_{ij})\mathbf{n}_{ij}. \quad \text{(S8)}$$

The parameter set appeared in the above-mentioned equations is given in Table S1. Additionally, the actual a_{ij} parameters in $\mathbf{F}_{ij}^{C}$ are compiled in Table S2.

References

1 R. D. Groot and P. B. Warren, Dissipative Particle Dynamics: Bridging the Gap between Atomistic and Mesoscopic Simulation, *J. Chem. Phys.*, 1997, **107**, 4423.

2 R. D. Groot and T. J. Madden, Dynamic simulation of diblock copolymer microphase separation, *J. Chem. Phys.*, 1998, **108**, 8713–8724.

Table S1. The values of the parameters used in DPD potentials.

Parameter	Value
σ	3
γ	4.5
r_s	0.86
C	4.0

Table S2. The a_{ij} parameters used in this work.

Pair	Nafion	SPEEK
A-B	24.44	22.55
A-C	49.54	41.14
B-C	49.05	36.54
A-W	107.91	116.80
B-W	116.05	88.69
C-W	11.60	12.65
A-A	25.0	25.0
B-B	25.0	25.0
C-C	25.0	25.0
W-W	25.0	25.0

Figure S1. Plots of time-dependent water connectivity for SPEEK (circle) and Nafion (a) (diamond) in the case of 30 vol% water content.

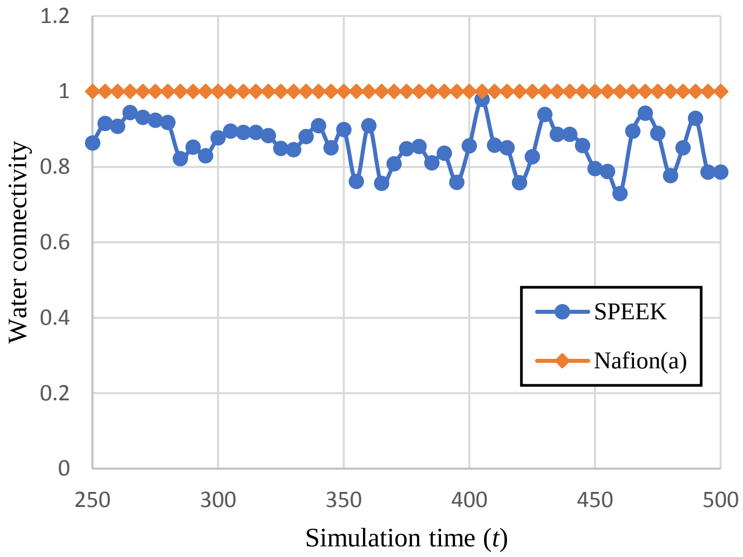

Supplement: RA-008-C8RA07428C-s001 [file RA-008-C8RA07428C-s001.pdf]
